# Engagement in an Interactive App for Symptom Self-Management during Treatment in Patients With Breast or Prostate Cancer: Mixed Methods Study

**DOI:** 10.2196/17058

**Published:** 2020-08-10

**Authors:** Marie-Therése Crafoord, Maria Fjell, Kay Sundberg, Marie Nilsson, Ann Langius-Eklöf

**Affiliations:** 1 Division of Nursing Department of Neurobiology, Care Sciences and Society Karolinska Institutet Stockholm Sweden

**Keywords:** engagement, adherence, mHealth, mobile app, cancer supportive care, symptom management, usage metrics, breast cancer, prostate cancer

## Abstract

**Background:**

Using mobile technology for symptom management and self-care can improve patient-clinician communication and clinical outcomes in patients with cancer. The interactive app Interaktor has been shown to reduce symptom burden during cancer treatment. It includes symptom assessment, an alert system for contact with health care professionals, access to self-care advice, and visualization of symptom history. It is essential to understand how digital interventions operate; one approach is to examine engagement by assessing usage and exploring user experiences. Actual usage in relation to the intended use—adherence—is an essential factor of engagement.

**Objective:**

This study aimed to describe engagement with the Interaktor app among patients with breast or prostate cancer during treatment.

**Methods:**

Patients from the intervention groups of two separate randomized controlled trials were included: patients with breast cancer receiving neoadjuvant chemotherapy (n=74) and patients with locally advanced prostate cancer receiving treatment with radiotherapy (n=75). The patients reported their symptoms daily. Sociodemographic and clinical data were obtained from baseline questionnaires and medical records. Logged data usage was retrieved from the server and analyzed descriptively and with multiple regression analysis. Telephone interviews were conducted with patients about their perceptions of using the app and analyzed using content analysis.

**Results:**

The median adherence percentage to daily symptom reporting was 83%. Most patients used the self-care advice and free text message component. Among the patients treated for breast cancer, higher age predicted a lower total number of free text messages sent (*P*=.04). Among the patients treated for prostate cancer, higher age (*P*=.01) and higher education level (*P*=.04), predicted an increase in total views on self-care advice, while higher comorbidity (*P*=.004) predicted a decrease in total views on self-care advice. Being married or living with a partner predicted a higher adherence to daily symptom reporting (*P*=.02). Daily symptom reporting created feelings of having continuous contact with health care professionals, being acknowledged, and safe. Being contacted by a nurse after a symptom alert was considered convenient and highly valued. Treatment and time-related aspects influenced engagement. Daily symptom reporting was perceived as particularly meaningful at the beginning of treatment. Requests were made for advice on diet and psychological symptoms, as well as for more comprehensive and detailed information as the patient progressed through treatment.

**Conclusions:**

Patient engagement in the interactive app Interaktor was high. The app promoted patient participation in their care through continuous and convenient contact with health care professionals. The predictive ability of demographic variables differed between patient groups, but higher age and a higher educational level predicted usage of specific app functions for both patient groups. Patients’ experience of relevance and interactivity influenced their engagement positively.

## Introduction

Treatments for cancer can lead to challenging symptoms, but most patients are managed as outpatients [1,2]. Patients’ own assessments of the occurrence and severity of symptoms and their concerns can inform and support health care professionals in identifying and assessing the potential risks associated with cancer treatment, leading to improved patient outcomes [3,4]. Interventions using mobile technology to support symptom monitoring and self-care among patients being treated for cancer have been shown to improve patient-clinician communication, improve symptom management and self-care ability, reduce symptom burden, and increase survival [4-6].

Even though apps to support symptom management for patients with cancer have increased, few feature evidence-based content or have been tested in rigorous trials [7,8]. Moreover, only some include interactive components, such as support for self-care and communication with health care professionals and peers for immediate clinical management [7,9-12]. A key aspect of digital interventions is to understand how they operate and how they can be enhanced by assessment of usage and user experiences [13-17].

We designed Interaktor, an interactive smartphone and tablet app, to support patient symptom management. The concepts of person-centered and participatory care inspired the development of the intervention [18,19]. The app is available in different versions tailored for patients during treatment for breast cancer, prostate cancer [20], and pancreatic cancer [21] and for older persons receiving home care [22]. The content of the different versions of the app was developed in an iterative process, which included reviews of literature focusing on symptoms and their management, clinical guidelines, interviews with patients, and interviews with health care professionals [21,23-25]. Detailed descriptions about the intervention outline and screenshots have been previously presented [20,26]. Interaktor includes four generic components: (1) Self-assessment through questions about symptoms and concerns, where patients report symptom occurrence, symptom frequency, and their distress level inspired by the Memorial Symptom Assessment Scale is a main component [27]. Patients also have the opportunity to report other symptoms with free text messages. The reports are immediately transferred via a secure server to health care professionals who can monitor patient reports in real time via a web interface. (2) A risk assessment model is included for symptoms that notifies nurses at the clinic by SMS text message when a high level of frequency or distress is reported for a symptom. There are two kinds of alerts: yellow and red. Yellow alerts require that a nurse contact the patient during the daytime, and red alerts require that a nurse contact the patient within 1 hour. (3) Evidence-based self-care advice and links to relevant webpages related to the assessed symptoms and other areas of concern are included. (4) Graphs showing reported symptom history for patients and health care professionals are also included. Interaktor can be used with Android and iOS, but the app is only available for research purposes. Logged data were stored on a separate secure server hosted by the health care company that developed the app.

Studies suggest that patients undergoing treatment for cancer make use of and appreciate opportunities to report symptoms to health care professionals when they are at home [26,28,29]. Mobile technology for health (mHealth) to support self-management has been linked to positive outcomes regarding physical as well as psychological symptoms in the context of cancer care [30,31]. An early system for remote symptom management during cancer treatment included symptom assessments twice a day, tailored advice, and access to informational webpages [5]; the study [5] found that patients differed markedly in the number of reports made and all patients viewed the webpages. Furthermore, modest problems with the technology were described, and patients rated improvements in the communication process with hospital staff and their satisfaction with care [32]. Systems for remote symptom management during cancer treatment have since demonstrated high acceptance [33] as well as long-term feasibility [34]. Most have been web-based and have involved symptom assessments from a home computer or clinic tablet [4,6,33,35].

Usage and user experiences of a web- or mobile-based intervention can be described by the concept of engagement [15]. Engagement is influenced by interconnected factors—some individual, such as demographics, skills, and understanding; some contextual, which include internet access and online environment; and some interventional, such as technical and design features [36]. Patients’ prior health behaviors and smartphone experience will affect how relevant and usable an intervention is perceived to be, which affects engagement, and persistent patient engagement is achieved if the intervention is perceived to be usable, relevant, helpful, and interactive [36]. Usage level compared to the intended usage is referred to as *adherence* [37]. The significance of adherence has gained increased recognition, since evidence has emerged that high levels of adherence positively correlate with improved outcomes [38]. However, within eHealth in general, levels of nonusage and dropout have been substantial, and achieving the desired level of patient adherence may be challenging [17]. Relating usage to intended use requires operationalization and rationalization [37]. Approaches to measuring adherence vary; reported methods include the number of log-ins, website exposure, and modules completed; these stem partly from diversity in the purpose and design of digital interventions but also demonstrate different views of the concept of adherence [38].

High levels of adherence to symptom reporting during cancer treatment have been observed in relation to clinic visits, but there is a lack of large-scale studies examining how patients undergoing treatment for cancer adhere to and perceive symptom monitoring and reporting via a mobile app [7,9].

Treatment for breast cancer consists of different approaches such as chemotherapy, surgery, and radiotherapy [39-41]. Chemotherapy can be administered as adjuvant after surgery, but in recent years, neoadjuvant chemotherapy, which is administered before surgery, has become more common [42]. Neoadjuvant chemotherapy is administered at the oncology clinic at different treatment intervals, depending on the cytotoxic drugs that are given [43]. Treatment for prostate cancer includes three main approaches: active surveillance, surgical treatment, and radiotherapy. These may be combined with antihormonal treatment. Radiotherapy for prostate cancer is administered every weekday at clinics [44]. In Sweden, patients remain at home between treatments. Patients with breast cancer undergoing neoadjuvant chemotherapy meet the physician before each treatment cycle, approximately every second or third week, depending on the chemotherapy regimen [43]. Patients with prostate cancer undergoing radiotherapy meet the physician before the start of radiotherapy and 6 months after completing radiotherapy [44]. Patients are assigned a contact nurse who is responsible for the patient’s care throughout the care chain and who the patient can contact during office hours in case of concerns related to the treatment [43,44]. Patients are also provided with telephone numbers for the oncology clinics, for making contact during office hours or during evenings, nights, and weekends.

As most patients undergoing neoadjuvant chemotherapy for breast cancer or radiotherapy for prostate cancer are treated on an outpatient basis, partially self-reliant management of symptoms is necessary. Several studies show decreased symptom burden [24,45], that patients appreciate using Interaktor, and that they feel secure [21,26]. The app is currently being evaluated in two randomized controlled trials that include patients with breast cancer and prostate cancer, with the hypothesis that using the app will improve symptom management, reduce symptom burden, and increase cost-effectiveness through a reduced consumption of health care services in comparison to standard care alone [20].

An understanding of how Interaktor is used and perceived, including the impacts of individual factors, is warranted in order to support the interpretation of the clinical effects of using the app during treatment [45]. Therefore, this study aimed to describe engagement with the Interaktor app among patients with breast and prostate cancer during their treatment.

## Methods

### Overview

This study included patients from the intervention groups of two separate randomized controlled trials: patients with breast cancer during neoadjuvant chemotherapy (ClinicalTrials.gov; NCT02479607) and patients with locally advanced prostate cancer during treatment with radiotherapy (ClinicalTrials.gov; NCT02477137). The study comprised logged data from patient reports and interviews with patients. Ethical approval was obtained from the Regional Ethical Review Board of Stockholm (registration no. 2013/1652-31/2 and 201712519-32).

### Setting and Sample

**Figure 1:**
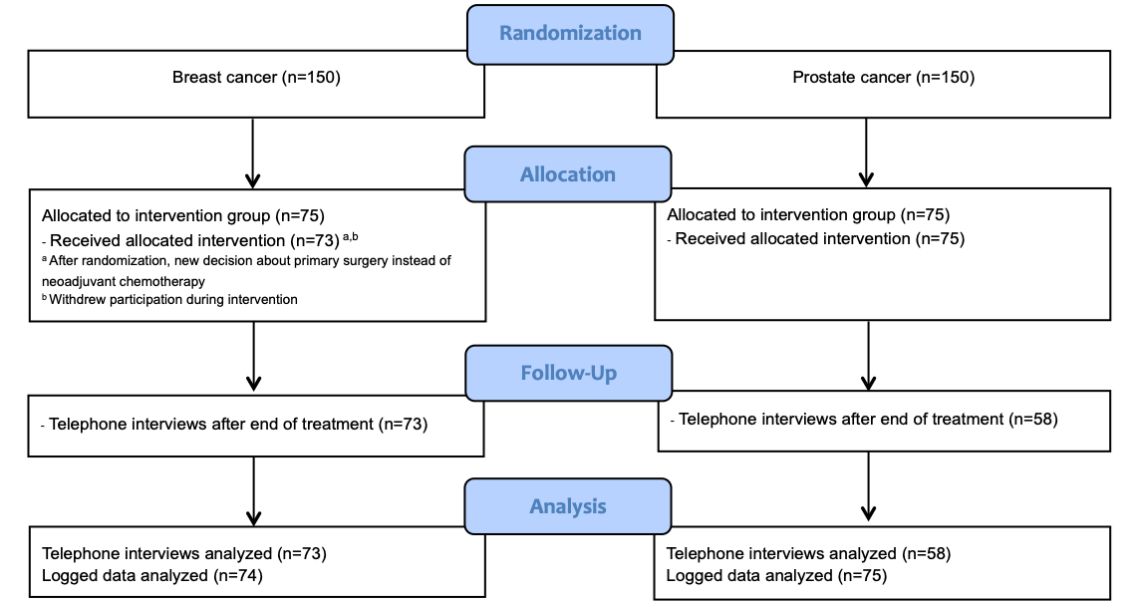
CONSORT diagram.

The patients were consecutively recruited at two university hospitals in Stockholm, Sweden. Patients with breast cancer or prostate cancer undergoing neoadjuvant chemotherapy and radiotherapy, respectively, who were able to speak and understand Swedish, who were presumed cognitively able to use a mobile app for symptom reporting, who agreed to participate, and who signed a written informed consent form were eligible. Of the 75 patients in the breast cancer group, 1 patient had a change of treatment to surgery instead of neoadjuvant chemotherapy, and 1 patient withdrew their consent to participate in the interview. In the prostate cancer group, 58 patients of the 75 patients participated in the interviews, due to organizational circumstances and difficulties reaching the patients after their treatment had ended (Figure 1).

### Study Procedure

Patients downloaded Interaktor onto their smartphone or tablet. Patients who did not own one were lent a smartphone by the research group (2 in the breast cancer group, 2 in the prostate cancer group). The patients also received an individual log-in for access to the content of the app. The patients received verbal and written instructions on how to use the app and were asked to report their symptoms daily on weekdays during treatment. Patients with breast cancer were asked to start using the app on their first day of neoadjuvant chemotherapy and continue until 2 weeks after treatment had ended—a total of approximately 18 weeks. Patients with prostate cancer started using the app on their first day of radiotherapy and continued until 3 weeks after treatment had ended—a total of approximately 9 weeks. All patients were informed that nurses at the clinic would survey and respond to alerts triggered on weekdays (8 AM to 4 PM). If patients needed support at other times of the day, they were instructed to contact the clinic according to the standard procedure. If a report had not been submitted before 2 PM, a notification was sent out to remind the patient to report. The breast cancer version of the app included a notification that was sent to the patient, suggesting the patient read self-care advice related to alerted symptoms. This function was not included in the prostate cancer version of the app.

### Data Collection

#### Participant Data

Sociodemographic data were obtained from baseline questionnaires. Clinical data and prevailing health status at the time of treatment start were collected from medical records. Health status was used to calculate the comorbidity score using the Charlson Comorbidity Index, which encompasses 19 medical conditions. Each condition has a score based on a relative risk of death within a year. The scores are totaled to yield the comorbidity score, with a range between 0 and 37. A higher value corresponds to greater comorbidity [46].

#### Logged Data

Data on app usage including symptom reports, triggered alerts, views of self-care advice, and free text messages sent, were made accessible to the researchers through encrypted Excel (2013; Microsoft Inc) files. At the time of the study, it was not possible for data on clicked links or viewed graphs to be logged for later retrieval.

#### Interviews

Telephone interviews were conducted with the patients shortly after the end of the use of the app, following a semistructured interview guide (Table 1) that we had developed, focusing primarily on usability, utility, and capturing participant feedback on the app (including all components). The interviews lasted between 10 and 20 minutes, during which the authors made notes of the answers in the template of the interview guide. Five of the patients with prostate cancer were individually interviewed in connection to more comprehensive interviews about participation in care. These interviews were recorded and transcribed verbatim, but only the text with respect to using the app was analyzed in this study.

**Table 1:** Interview guide.

	Question	Follow-up question
1	What was it like to report in the app? (in general)	Easy/difficult? Advantages/disadvantages?
2	What was it like to report your symptoms?	Easy/difficult? Absence of symptom to report?
		Relevant symptom questions and reporting frequency?
3	Have you used the self-care advice? (yes/no)	If yes, your experience? Relevant self-care advice? Have you used the links?
		If no, have you searched for information elsewhere?
4	How have you experienced that the technology has worked?	The log-in procedure? Mobile coverage?
5	Have you used the graphs to follow your symptom history? (yes/no)	If yes, in which way? How did you experience the graphs?
		If no, why?
6	Were you called sometime by a nurse? (yes/no)	If yes, was it after an alert?
7	How did you experience being called by a nurse after an alert?	—
8	Is there anything else you want to add?	Is there anything in the app that you have lacked?

### Statistical Analyses

Data management was performed using Excel, and statistical analyses were carried out using SPSS statistical software (version 24.0; IBM Corp). Differences in demographic and clinical characteristics between the two groups were analyzed using two-tailed independent *t* test, Fischer exact test, and the chi-square test. Usage was analyzed with descriptive and inferential statistics on the variables reports sent, alerts triggered, self-care advice section views, and free text messages sent; group median values were calculated and are reported. The intended use was measured by daily symptom reporting during weekdays. Adherence to daily reporting was calculated as the number of weekday reports (excluding multiple daily reports) divided by the total number of reportable weekdays for each patient.

Multiple regression analysis was conducted (using the enter method) to see if the independent variables (predictors) age, comorbidity, marital status, and education level predicted usage of the app. The usage variables (dependent variables) were adherence to daily reporting as intended, total number of alerts triggered, total views on self-care advice, and total number of free text messages sent. Since all of the usage variables were positively skewed, these were normalized using a natural logarithm transformation [47]. Level of significance was determined as *P*<.005.

### Qualitative Analysis

The interview notes were analyzed by conventional content analysis [48]. First, two authors read the complete interview notes from each group (breast cancer and prostate cancer) individually, to grasp the entire data. Both authors compiled a data sheet for each group with the patients' answers. The data sheets were reviewed repeatedly, and initial codes were derived. Thereafter, all authors reviewed both sets of data sheets and codes. Due to substantial similarities of codes in the two groups (breast cancer and prostate cancer), one data set containing all coded responses (with credentials) was assembled. Subsequently, all text material were analyzed. In an iterative process, the codes were sorted based on similarity into subcategories and combined based on content into overarching categories. Some exemplifying quotes from the patients’ statements are presented in the Results section. During the analytical process, all authors met continually to discuss and come to a consensus.

## Results

### Sample Characteristics

The patients’ sociodemographic (Table 2) and clinical data (Table 3) are presented below. Patients in the breast cancer group were significantly younger than the patients in the prostate cancer group (*P*<.001). Moreover, the patients in the breast cancer group had a statistically significant lower comorbidity score (*P*<.001) and a higher self-reported education level (*P*=.005).

**Table 2:** Sociodemographic characteristics at baseline of patients with breast cancer and prostate cancer.

Characteristics			Breast cancer (n=74)		Prostate cancer (n=75)		Test statistic		*P* value
Age (in years), median (range)			47 (27-73)		72 (44-81)		–15.127^a^		<.001^a^
**Marital status, n (%)**			74 (100)		71 (100)		1.204 (2)^b^		.57^b^
	Married/cohabitant	58 (78)		53 (74)					
	Living apart	3 (4)		6 (9)					
	Single	13 (18)		12 (17)					
**Highest education level, n (%)**			74 (100)		71 (100)		10.627 (2)^c^		.005^c^
	University	50 (68)		30 (42)					
	Secondary school	18 (24)		25 (35)					
	Primary school	6 (8)		16 (23)					
**Occupation, n (%)**			74( 100)		69 (100)		61.330 (2)^b^		<.001^b^
	Working	57 (77)		22 (32)					
	Sick leave	12 (16)		0 (0)					
	Retired/unemployed	5 (7)		47 (68)					

^a^Mann-Whitney *U* test.

^b^Fischer exact test (*df*).

^c^Chi-square (*df*).

**Table 3:** Clinical characteristics at baseline of patients with breast cancer and prostate cancer.

Characteristics		Breast cancer (n=74)	Prostate cancer (n=75)	Test statistic	*P* value
Charlson Comorbidity Scale score^a^, median (IQR)		1.0 (1)	3.0 (1)	5082.5^b^	<.001^b^
PSA^c^ (at start of treatment), median (range)		N/A^d^	5.3 (0-53)		
**Disease stage (TNM^e^), n (%)**					
	T1	N/A	1 (1)		
	T1C	N/A	17 (23)		
	T2	N/A	11 (15)		
	T2A	N/A	1 (1)		
	T2B	N/A	7 (9)		
	T2C	N/A	7 (9)		
	T3	N/A	20 (27)		
	T3A	N/A	1 (1)		
	T3B	N/A	8 (11)		
	Missing	N/A	2 (3)		
**Histologic grade (Elston-Ellis), n (%)**					
	Intermediate grade 2	23 (31.1)	N/A		
	High grade 3	41 (55.4)	N/A		
	Unknown	10 (13.5)	N/A		
**Tumor characteristics^f^, n (%)**					
	HER2+ ER+ PR+	9 (12.2)	N/A		
	HER2+ ER+ PR–	7 (9.5)	N/A		
	HER2+ ER– PR–	13 (17.6)	N/A		
	HER2– ER+ PR+	16 (21.6)	N/A		
	HER2– ER+ PR–	7 (9.5)	N/A		
	HER2– ER– PR+	1 (1.4)	N/A		
	Triple negative	21 (28.4)	N/A		
**Proliferation rate (Ki-67), n (%)**					
	≥ 20 %	72 (97.3)	N/A		
	< 20 %	2 (2.7)	N/A		
**Type of chemotherapy, n (%)**					
	Anthracyclines, alkylators	3 (4.1)	N/A		
	Anthracyclines, alkylators, taxanes	37 (50.0)	N/A		
	Anthracyclines, alkylators, antimetabolites, taxanes	9 (12.2)	N/A		
	Antimetabolites, alkylators	2 (2.7)	N/A		
	Taxanes	11 (14.9)	N/A		
	Taxanes, alkylators	1 (1.4)	N/A		
	Taxanes, trastuzumab emtansine	2 (2.7)	N/A		
	Trastuzumab emtansine	9 (12.2)	N/A		
Gleason score, median (range)		N/A	7 (6-9)		
Number of radiotherapy treatments, median (range)		N/A	25 (25-29)		
Number of neoadjuvant chemotherapy treatments, median (range)		6.0 (2-15)	N/A		
**Antihormonal treatment, n (%)**					
	Yes	N/A	55 (73)		
	No	N/A	20 (27)		
Treatment duration (in weeks), median (range)		15 (3-26)	5 (5-6)		

^a^A higher value corresponds to greater comorbidity; the score ranges between 0-37.

^b^Mann-Whitney *U* test.

^c^PSA: Prostate-specific antigen.

^d^N/A: not applicable.

^e^TNM: Classification of malignant tumors.

^f^Breast cancer cell human epidermal growth factor receptor (HER2), estrogen receptor (ER), and progesterone receptor (PR) status.

### Logged Data

**Figure 2:**
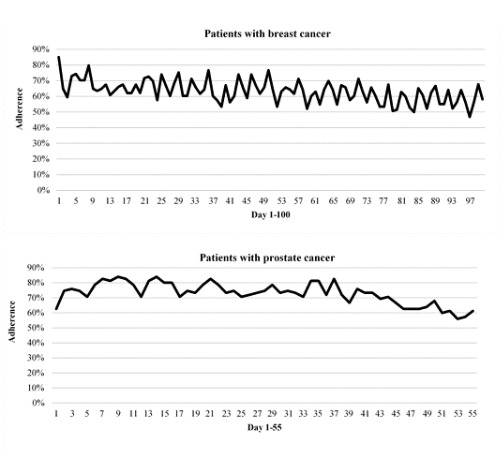
Adherence to symptom reporting over time.

All patients reported with the app at least once during the study period. Due to differences in treatment schedules, the reporting period in the breast cancer group ranged from 22 to 183 days (median 106, IQR 7). The reporting period in the prostate cancer group ranged from 54 to 89 days (median 63, IQR 11). Median adherence in the breast cancer group was 83% (IQR 36%). In the prostate cancer group, the median adherence percentage was also 83% (IQR 34%). Graphs of adherence patterns over time show that the level of adherence remained stable over time, although it dropped somewhat after day 49 in the breast cancer group and day 39 in the prostate cancer group (Figure 2).

In the breast cancer group, 96% (71/74) of patients triggered at least one alert during the study period; in the prostate cancer group, 72% (54/75). Patients in the breast cancer group triggered a median of 7 alerts (IQR 21, range 1-210). In the prostate cancer group, the median number of alerts triggered was 2 (IQR 9, range 1-60). The distribution of yellow and red alerts was 86% yellow to 14% red in the breast cancer group and 90% yellow to 10% red in the prostate cancer group.

Among patients in the breast cancer group, 100% (74/74) viewed self-care advice at least once. Patients in the breast cancer group viewed a median of 11 (IQR 15) self-care advice topics at least once during the study period, out of 17 self-care advice available. The total number of views was 1075, and 34% (362/1075) were views made after the patient was notified with the suggestion to read related self-care advice after an alert had been triggered. In the prostate cancer group, 87% (65/75) of patients viewed self-care advice at least once and the median number of various self-care advice topics viewed at least once was 5 (IQR 11) out of 16 self-care advice available. The total number of self-care advice views by patients in the prostate cancer group was 697.

Most of the patients used the free text function at least once: 93% (69/74) in the breast cancer group and 75% (56/75) in the prostate cancer group. The free text messages were mainly about symptoms, requesting or declining contact, care-related information, and issues linked to the app or to reporting. There was a variation in how the function was used, some patients wrote short, condensed messages while some wrote longer, richer descriptions.

### Patient Characteristics as Predictors of App Usage

In the breast cancer group none of the multiple regression models were statistically significant (Table 4). Higher age predicted a lower total number of free text messages sent (*P*=.04). In the prostate cancer group the multiple regression model showed that the total number of views on self-care advice was statistically significant (*F*_4,70_=3.811, *P*=.007, adjusted R^2^=.132) (Table 5). Higher age (*P*=.01) and higher education level (*P*=.04) predicted a higher total number of views of self-care advice. Higher comorbidity score predicted fewer self-care advice views (*P*=.004). Furthermore, being married or cohabitating predicted a higher adherence to daily symptom reporting (*P*=.02) (Table 5).

**Table 4:** Multiple regression in patients with breast cancer (n=74).

Dependent and independent variables			B^a^		SE^b^		β^c^		95% CI				*P* value^d^		Adj^e^ R^2^		*P* value^f^
								Lower		Upper							
**Adherence to daily reporting as intended**															<.001		.72
	Age	–0.006		0.010		–.080		–.027		.015		.56					
	Comorbidity	0.094		0.099		.129		–.103		.291		.35					
	Marital status	0.039		0.230		.020		–.420		.497		.87					
	Educational level	0.224		0.208		.132		–.191		.639		.29					
**Total number of alerts**															.038		.16
	Age	–0.027		0.015		–.243		–.056		.003		.07					
	Comorbidity	–0.087		0.140		–.082		–.367		.193		.54					
	Marital status	–0.387		0.326		–.137		–1.04		.264		.24					
	Educational level	–0.116		0.296		–.046		–.705		.474		.70					
**Total views on self-care advice**															<.001		.46
	Age	–0.007		0.011		–.084		–.028		.015		.54					
	Comorbidity	–0.101		0.102		–.134		–.303		.102		.33					
	Marital status	0.131		0.236		.066		–.340		.603		.58					
	Educational level	0.093		0.214		.053		–.334		.520		.66					
**Total number of free text messages**															.070		.07
	Age	–0.027		0.013		–.295		–.052		–.001		.04					
	Comorbidity	0.036		0.114		.044		–.191		.264		.75					
	Marital status	–0.030		0.271		–.013		–.571		.511		.91					
	Educational level	0.326		0.238		.165		–.149		.800		.18					

^a^B: unstandardized coefficient.

^b^SE: standard error of the unstandardized coefficient.

^c^β: standardized coefficient.

^d^*P* value for the independent variable.

^e^Adj: adjusted for the multiple regression model.

^f^*P* value for the multiple regression model.

**Table 5:** Multiple Regression in patients with prostate cancer (n=75)

Dependent and independent variables		B^a^	SE^b^	β^c^	95% CI		*P* value^d^	Adj^e^ R^2^	*P* value^f^
					Lower	Upper			
**Adherence to daily reporting as intended**								.027	.21
	Age	–0.001	0.015	–.008	–.030	.028	.96		
	Comorbidity	0.020	0.077	.038	–.134	.173	.80		
	Marital status	0.431	0.181	.277	.071	.792	.02		
	Educational level	–0.026	0.165	–.018	–.355	.304	.88		
**Total number of alerts**								< .001	.66
	Age	–0.011	0.026	–.067	–.062	.040	.66		
	Comorbidity	0.032	0.134	.036	–.236	.300	.81		
	Marital status	0.482	0.316	.182	–.148	1.11	.13		
	Educational level	0.082	0.289	.033	–.494	.657	.78		
**Total views on self–care advice**								.132	.007
	Age	0.057	0.022	.368	.013	.101	.01		
	Comorbidity	–0.347	0.116	–.419	–.579	–.115	.004		
	Marital status	0.019	0.273	.008	–.526	.565	.94		
	Educational level	0.531	0.250	.232	.032	1.03	.04		
**Total number of free text messages**								< .001	.84
	Age	–0.008	0.024	–.052	–.055	.039	.74		
	Comorbidity	–0.029	0.125	–.035	–.278	.220	.82		
	Marital status	0.229	0.293	.094	–.356	.814	.44		
	Educational level	–0.161	0.268	–.071	–.696	.374	.55		

^a^B: unstandardized coefficient.

^b^SE: standard error of the unstandardized coefficient.

^c^β: standardized coefficient.

^d^*P* value for the independent variable.

^e^Adj: adjusted for the multiple regression model.

^f^*P* value for the multiple regression model.

### Perceptions of Using the Interaktor App

#### Overall

Although all patients were enthusiastic about contributing to the study and to the evaluation and development of the app, some patients noted that they did not recall using the app in detail. Analysis of the interviews resulted in three overarching categories: user friendliness, interaction with the health care professionals, and support for self-care.

#### User Friendliness

Nearly all patients stated that the app was easy to use and that it took little time to learn how to use it. Few experienced technical problems.

Reporting went quickly, and the app was described as a fast and comfortable way to get support and help. Most patients agreed that the app content was relevant and that the symptoms included in the app covered most symptoms they experienced during their treatment. Only some patients wanted to report symptoms that were not included in the app, such as headache and weight gain.

Symptom reporting was often described as more meaningful and interesting in the beginning when symptoms were new and there was a feeling of apprehension and insecurity of what the treatment would entail. When symptoms had become a fact of everyday life, reporting them via the app daily did not always feel necessary. A few patients described daily symptom reporting as something they perceived as a negative reminder of illness, especially during times when they felt alright.

It has been very easy and convenient….The app is easy to use….When you feel ill it is a security, but if you feel good it is a negative reminder that you are sick.

BI-10

Remembering to report symptoms was sometimes perceived as difficult, particularly when patients felt well. Incorporating or establishing a routine around reporting facilitated recollection, as did the automatic reminder notifications, which were greatly appreciated. Some patients requested the ability to adjust the reporting time according to personal preferences.

In the breast cancer group, daily reporting could sometimes be stressful and difficult, especially when they felt ill or lacked energy; nevertheless, most patients considered reporting to be more necessary at those times. Furthermore, memory impairment was often described as linked to the cognitive side effects of treatment and some mentioned that graphs were difficult to interpret because the text was too small. In the prostate cancer group, some patients did not notice or use the self-care advice, links, or graphs due to lack of experience with mobile apps or due to forgetfulness. Patients suffering from comorbidities that had symptoms similar to the questions included in the app perceived reporting as difficult to answer, given the available responses.

#### Interaction With Health Care Professionals

Reporting symptoms generated a feeling of having continuous contact with health care professionals. It also created feelings of being safe, monitored, acknowledged, involved, and cared for. Most patients described being called by a nurse after an alert in positive terms and said it decreased the need for contacting the oncological clinic through other channels. They said that it was a great benefit to not have to call, leave a message on the answering machine, be put on hold, or have to search for the right phone number. On a few occasions, patients were not contacted after alerts.

I felt safe reporting every day....It was excellent….I noticed, before logging off, that I would be called...A huge security….Having the app and a continuous access to help has made me feel better.

BI-44

In the breast cancer group, some patients expressed that alerts should be monitored and responded to around the clock, and several wanted the possibility to choose themselves whether a nurse should call them. Some preferred calling health care professionals, especially when they felt very ill. A small number of patients in the breast cancer group perceived themselves as nagging or bothering the nurses when alerts were triggered.

Some patients indicated a wish to continue reporting after the trial period had ended. They described feelings of being alone when the treatment ended, and they no longer met their health care professionals regularly. At that time, the value of symptom reporting was perceived to increase.

The free text function was generally appreciated and was perceived as useful for reporting additional symptoms and information to the nurse.

#### Support for Self-Care

The patients perceived the app as supportive during their treatments and described symptom reporting in terms of diary keeping. Reporting symptoms encouraged and supported reflection on their well-being and made patients more aware of symptoms and what they should observe for.

A majority of the patients perceived the self-care advice as valuable, applicable, and informative, especially when a symptom first occurred. The self-care advice gave answers on how to perform self-care to relieve or manage symptoms for themselves. Reading the advice also gave them an idea of what was normal and what they should expect during treatment. There were requests to add more comprehensive information on psychological symptoms and dietary advice. The patients in the breast cancer group described the graphs as useful for comparing symptoms over time and detecting patterns in the symptoms related to the cytotoxic treatment intervals. This could facilitate the planning of activities and enable them to do things during days they felt well. The patients in the prostate cancer group often commented that the graphs enabled them to monitor their well-being by displaying when symptoms increased or decreased and also helped them discern that many days were trouble free.

It was said about the symptom history graphs—

It was fun to see that it was getting better… and it trailed with how you perceived to be feeling.

PI-187

I did not follow them….I want to move on….Now it is just forward ahead.

PI-143

The patients in the breast cancer group perceived the links as useful for gaining further or in-depth information and support. Patients in the prostate cancer group were more likely to describe the self-care advice, links, and graphs as superfluous when they experienced mild or less persistent symptoms.

## Discussion

### Principal Results

The findings of this study show that the app was largely used as intended and appreciated by patients undergoing treatment for breast cancer and prostate cancer. The app gave the patients a feeling of assurance by offering a convenient method to contact their health care professionals and the security of being monitored via the symptom reports. Furthermore, the app promoted self-care by facilitating self-monitoring and learning about symptoms.

In this study, adherence to daily symptom reporting in the app was 83%. This is high in comparison to a review [49] in which it was concluded that around half of participants in web-based health interventions for chronic conditions and lifestyle and mental health management adhered to the interventions. Plausible explanations for the high adherence to the Interaktor app are that the app was interactive and easily available on a smartphone or tablet when compared to a computer-based system. Previous research [49,50] shows that intervention characteristics such as intended usage frequency, updates, and persuasive design increase patients’ adherence to web-based interventions. Moreover, the app may reflect patients’ need for frequent, continuous contact with health care professionals during treatment for cancer [51].

Most patients perceived the content of the Interaktor app as relevant, and the fact that Interaktor was developed in collaboration with patients and health care professionals is likely to have contributed to this result. Two previous studies [21,26] of the Interaktor app among patients treated for cancer yielded results in line with this study, both in terms of adherence levels above 80% and interviews revealing that patients appreciate and perceive the app as supportive in their symptom management.

There was a temporal aspect to how patients perceived using the app. At the beginning of treatment, when the situation was new or they experienced a new symptom, daily symptom reporting was considered especially meaningful. Later, as patients became more experienced and familiar with their symptoms, some noted a lower inclination to report each day. Also, patients commented that after some time, as they acquired general knowledge about the disease and treatment, they felt a desire for more individualized and in-depth information than that contained in the app.

The observation that memory impairment and feeling ill influenced engagement adversely contrasts with a study [52] that showed that increased use of a web-based symptom management system was predicted by higher levels of symptom distress among men with prostate cancer, and it may be of value to investigate the effect of symptom burden on app usage in future studies.

The interviews indicated that some patients with comorbidities felt the need to add clarifications to responses available in the self-assessment form by free text. This finding warrants further study, considering the expanding community with multiple conditions, and it contrasts somewhat from the findings of a study [52] that showed that increased use of the system was associated with the absence of comorbidities.

The findings described above are in line with a conceptual model where perceived credibility and personal relevance influence engagement [36]. This study accumulates existing evidence, which imply the need to develop cancer-supportive digital interventions that are interactive and tailored [53]. Tailoring can be performed by the individual patient before the intervention (pretailoring), by preference to promote autonomy (self-tailoring), and within-person as health status or needs evolve [54]. In future technological development of the app, it might be useful to integrate an option for the individual patient to add or exclude symptoms or concerns in the self-assessment component to make it more person-centered. Expanding the interactive features in Interaktor by adding or updating information as patients progress through treatment may also be a way to maintain and enhance patients' experience of personal relevance [36].

The adjusted R^2^ values in the regression models in this study were low, indicating that none or only one predictor was correlated to each dependent variable. The only variable tested that predicted usage for both groups was age; higher age predicted a decrease of the total number of free text messages sent in the breast cancer group, while a higher age predicted more self-care advice views in the prostate cancer group. It has previously been suggested that demographic variables may be too broad to indicate usage motives and preferences for mHealth [55]. Higher education level predicted usage in the prostate cancer group, specifically, predicting a higher number of views of self-care advice. Furthermore, in the prostate cancer group, being married or cohabiting predicted higher adherence to daily reporting as intended than that of patients who were single. These findings are in line with theory as well as research suggesting that social support and education level influence the adoption and usage of web-based interventions [17,56]. Both social support and higher education level have been associated with a significant increase in engagement [15].

In the breast cancer group, it was suggested by patients that they should be responsible for contacting health care professionals. This finding is noteworthy, as it pertains to patients’ sense of control and self-reliance. From the perspective of a review on person-centered participation [57], this may signify a patient with confidence in their own experience-based knowledge and expertise, voicing a willingness for increased responsibility and management of their care. This is an essential precondition if we are to achieve equality and partnership among health care professionals and patients, but it is always preceded by phases of dialogue, knowledge building, and information sharing [57]. Previous studies have demonstrated that being female, having a higher education level, and being younger are predictive for preferring a more active role in care [58,59].

### Strengths and Limitations

Analysis of usage metrics enables a systematic assessment of, and insight into, patients’ exposure to and behavior throughout an intervention relating to frequency, depth, and breadth of use [17] but cannot disclose how use was perceived [60]. On the other hand, interviews or self-reports are associated with a risk of social desirability and challenges in communication, such as discrepancies in researcher and respondent terminology [60].

To counteract the limitations of each method, a combination was used in this study. Below are two examples of findings that reinforce how relying on only one source of data to study engagement is not sufficient. In the interviews, patients described aspects that were perceived to influence their engagement, which were not visible in the logged data. Patients stated that remembering to report daily was sometimes difficult, and moreover, patients noted that daily symptom reporting was perceived as more meaningful in the beginning. But the logged data show that adherence to daily symptom reporting is high and stable throughout the time of the intervention as adherence patterns do not decrease markedly over time. Relying on either one of the methods would have resulted in a less comprehensive account of patient engagement.

It may be a limitation that all the interviews took place after the intervention was completed and collecting data during the intervention as well as afterward may have added additional insights relating to usability and learning [61]. Symptom graphs and self-care advice features went undiscovered by a small number of patients in the significantly older prostate cancer group, indicating that enhanced patient training and further reviews of usability may be profitable in future studies. Research contributing to an understanding of how adherence can be promoted is starting to emerge [50,61,62]. However, a comparison of adherence levels between studies is debatable due to limited consistency in how adherence is reported [37,38]. The results of this study provide valuable knowledge, as it relates both to the patients’ actual usage versus intended use of the intervention and to how the patients thought about using it. The strength of this study consolidating investigations of two distinct patient groups is somewhat limited as the two versions of Interaktor slightly differed. The prostate cancer version of the app was developed and clinically trialed before the planning of this study and did not have the function that automatically prompted reading of specific self-care advice based on symptom reports. It is also a limitation that the use of links and graphs were not logged. Both are, in a sense, reflective of a learning curve in innovation research and will be taken into consideration in forthcoming studies.

### Conclusions

High patient engagement in the Interaktor app was achieved. Using the app promotes self-care by facilitating self-monitoring and timely advice. Furthermore, it provides assurance through continuous and convenient contact with health care professionals.

The study supports the notion that interactivity enhances patients’ feelings of personal relevance and thus increases engagement. The predictive ability of demographic variables differed between patient groups, but higher age and a higher educational level predicted the usage of specific app functions for both patient groups.

Taken together, the findings suggest a role for the use of an interactive app, such as Interaktor, to promote patients' participation in their care. The findings may have relevance for outpatients undergoing other cancer treatments associated with a risk of toxicities.

## Abbreviations

IQR: interquartile range
mHealth: mobile technology for health
