# Correction: Engagement in an Interactive App for Symptom Self-Management during Treatment in Patients With Breast or Prostate Cancer: Mixed Methods Study

**DOI:** 10.2196/33140

**Published:** 2021-10-12

**Authors:** Marie-Therése Crafoord, Maria Fjell, Kay Sundberg, Marie Nilsson, Ann Langius-Eklöf

**Affiliations:** 1 Division of Nursing Department of Neurobiology, Care Sciences and Society Karolinska Institutet Stockholm Sweden

In “Engagement in an Interactive App for Symptom Self-Management during Treatment in Patients With Breast or Prostate Cancer: Mixed Methods Study” (J Med Internet Res 2020;22(8):e7058), 5 errors were noted.

In the originally published article, the following sentence was included in the Abstract under the “Results” section:

Among the patients treated for breast cancer, higher age predicted a higher total number of free text messages sent (*P*=.04).

This sentence has been corrected to:

Among the patients treated for breast cancer, higher age predicted a lower total number of free text messages sent (*P*=.04).

In the originally published article, the following sentence was included in the Abstract under the “Conclusions” section:

The predictive ability of demographic variables differed between patient groups, but higher age and a higher educational level predicted higher usage of specific app functions for both patient groups.

This sentence has been corrected to:

The predictive ability of demographic variables differed between patient groups, but higher age and a higher educational level predicted usage of specific app functions for both patient groups.

In the originally published article, the following sentence was included in the “Results” section under the paragraph “Patient Characteristics as Predictors of App Usage”:

Higher age predicted a higher total number of free text messages sent (*P*=.04).

This sentence has been corrected to:

Higher age predicted a lower total number of free text messages sent (*P*=.04).

In the originally published article, the following sentence was included in the “Discussion” section under the paragraph “Principal Results”:

The only variable tested that predicted usage for both groups was age; higher age predicted an increase of the total number of free text messages sent in the breast cancer group, while a higher age predicted more self-care advice views in the prostate cancer group.

This sentence has been corrected to:

The only variable tested that predicted usage for both groups was age; higher age predicted a decrease of the total number of free text messages sent in the breast cancer group, while a higher age predicted more self-care advice views in the prostate cancer group.

In the originally published article, the following sentence was included in the “Conclusions” section:

The predictive ability of demographic variables differed between patient groups, but higher age and a higher educational level predicted higher usage of specific app functions for both patient groups.

This sentence has been corrected to:

The predictive ability of demographic variables differed between patient groups, but higher age and a higher educational level predicted the usage of specific app functions for both patient groups.

The correction will appear in the online version of the paper on the JMIR Publications website on October 13, 2021, together with the publication of this correction notice. Because this was made after submission to PubMed, PubMed Central, and other full-text repositories, the corrected article has also been resubmitted to those repositories.

